# Items

**Published:** 1883-08

**Authors:** 


### Items.

THE following is, as nearly as type can reproduce it, a fac-simile of a postal card recently received by the Illinois State Board of Health :

Fieldon Ill 5-12-83.
John H Rauch M D. Sir. you sti-
ll persist in trying to freighten
our Graduates, like Springer of
Hardin. who have attended two
full, long terms in our School &
Honorably graduated. I look
upon you, as the chief, of a set of
Dastard Bigots & villians & the
sooner you arrest Springer the so-
oner you will get through with it.
Yours. L C. Washburn. President of
the St Louis Eclectic Medical College.

The " Dastard Bigots & villains," who compose the Illinois State Board of Health, decline to recognize the St. Louis Eclectic, and "freighten" its alleged graduates by refusing to issue them certificates entitling to practice. *Hinc illæ.*

On the 14th of March last a certificate was issued by the State Board of Health, entitling one Julius Nothhelfer, of 701 Pine street, St. Louis, to practice medicine and surgery in Illinois. The application for this certificate was based upon an affidavit that Nothhelfer was a graduate of the St. Louis Medical College, and this was corroborated by a letter from a member of the faculty of that institution, in which he says: "I can sincerely indorse Dr. Julius Nothhelfer, a graduate of the St. Louis Medical

College at the session of 1879-80, as to his moral and professional character and standing." In his first letter, March 7, Nothhelfer states that he intends "to practice in a country town in Illinois."

On the 24th of the same month, a similar certificate was issued to one Joseph Fitzgerald, also of 701 Pine street, St. Louis, and this was granted upon an affidavit that Fitzgerald graduated from the Royal College of Snrgeons, Ireland, in 1871. The affidavit was accompanied by a number of letters "bearing testimony to the capabilities of Dr. Fitzgerald as a medical man * * * eminently suited to take charge of any institution requiring a courteous and skilful medical officer." These were all written in Dublin, none later than 1874, and as this was not deemed recent enough by the secretary, a rather cautiously worded letter was forwarded by Fitzgerald from a prominent St. Louis physician, its reticence being plausibly explained by Fitzgerald on the ground of the latter being a stranger in that city. Fitzgerald requested that his certificate be sent to Belleville, St. Clair county, where he proposed to practice.

Last week the attention of Dr. Rauch, secretary of the State Board, was attracted by a large three column, four-page circular, headed "The Great Surgeons, Drs. Nothhelfer and Fitzgerald, late of London, Eng., graduates of King and Queen's college, will be at the Commercial Hotel, 100 to 104 Lake street, Chicago, for one month. Terms for treatment, cash. Charges, from $10 to $500, owing to the requirements of the case." In this circular, they not only promise to cure every imaginable disease, but offer $500 reward for an incurable case. Inferentially, it is the bulletin of the "St. Louis Medical and Surgical Institute," an establishment which, it is safe to say, has no tangible existence ; but which, in this sheet at least, is "equipped with the most perfect surgical instruments and appliances, furnishes the most approved methods of treatment, and treats successfully the great majority of the diseases and deformities considered and given up as incurable." Everything is done by "new methods of treatment, new and recently invented instruments, and new and recently discovered remedies." Some of the passages are unique even in quack literature.

Of course correspondence is solicited after the usual method, and for the usual purpose of the infamous fraternity; consultations are free, and every imaginable device is employed to entrap the victim. What is meant by "consultations free" was tested yesterday, when they offered to visit and prescribe for an imaginary patient gratis, but would charge $10 for the medicines.

The case is such a flagrant one, and comes so clearly within the authority of the Board, unhampered by the ten years' exemption clause, which shields the doctors Jameses, Keans, Clarks and others, that the secretary laid the fact at once before the individual members, and received their prompt authorization to revoke the certificate. After to-day, therefore, any professional act performed by "the great surgeons, Drs. Nothhelfer and Fitzgerald," in the State of Illinois, will subject them to fine and imprisonment in the county jail.

The foregoing was published in the Chicago and St. Louis papers of Wednesday A. M., May 2. Before noon of that day the street distributors of the circular were arrested, and within 24 hours Nothhelfer and Fitzgerald had "folded their tents," etc. It is understood they went to Milwaukee.

DR. JAMES NEVINS HYDE, of this city, is at the sea-shore. He will return to Chicago by the 1st of September, immediately after the annual meeting of the American Dermatological Association at Lake George.

ECSTASY.

Several physicians of Paris, and especially those, who make a study of nervous diseases, have been lately much concerned about a case of ecstasy, presented by a woman taken in the Beaujon Hospital. She was found by the police lying in a seat in the Champs Elysée completely insensible. Taken to the station, Dr. Pinel could not awaken her, although he used every means generally adpated to such an end. The patient is in an advanced state of pregnancy, has the eyes wide open, keeps the mouth closed, does not speak and eats little and rarely.

GONORRHŒAL SCIATICA.

There seems to exist a very interesting relation between gonorrhœa and the development of sciatic neuralgia. Becchini reports in *Lo Sperimentale* the history of two patients taken with acute pains along the course of the sciatic nerves during the acute stages of gonorrhœa. All the usual remedies used for sciatica were tried without success. Relief was not obtained until the complete cure of the gonorrhœa, treated with the usual remedies.—*Le Praticien.*

DR. ROSWELL PARK, of this city, is about to remove to Buffalo, where he will engage in the practice of surgery. We can ill-afford to lose his name from the list of the profession in Chicago.

DR. CHAS. GILMAN SMITH is at the east. He is expected to return in August.

